# Smoking trajectory and cancer risk: A population-based cohort study

**DOI:** 10.18332/tid/152137

**Published:** 2022-08-26

**Authors:** Minh N. Luu, Minji Han, Tra T. Bui, Phuong Thao T. Tran, Min-Kyung Lim, Jin K. Oh

**Affiliations:** 1 Department of Cancer Control and Population Health, Graduate School of Cancer Science and Policy, National Cancer Center, Goyang-si, Republic of Korea; 2 Department of Research Methodology and Biostatistics, School of Preventive Medicine and Public Health, Hanoi Medical University, Hanoi, Vietnam; 3 Division of Cancer Prevention, National Cancer Control Institute, National Cancer Center, Goyang-si, Republic of Korea; 4 Department of Social and Preventive Medicine, College of Medicine, Inha University, Incheon, Republic of Korea

**Keywords:** population-based study, cancer risk, smoking trajectory

## Abstract

**INTRODUCTION:**

Smoking behavior can change with time and lead to different health outcomes. This study explored the trajectory of smoking and its relationship with cancer incidence and mortality among Korean male adults.

**METHODS:**

We used 2002–2018 data from the National Health Insurance Service (NHIS). Smoking status was repeatedly measured in four waves of general health examinations provided by the NHIS between 2002 and 2009. Cancer incidence and mortality were tracked from 2010 to 2018. Trajectory analysis was used to identify the patterns of smoking. The hazard ratio was calculated using Cox proportional regression models.

**RESULTS:**

For the 2448548 men (≥20 years), 137788 cases of cancers and 41146 cancer deaths were found. We identified six trajectory groups: never smokers, former smokers, new current smokers, decreasing light smokers, steady moderate smokers, and steady heavy smokers. All smoking groups had an increased risk of cancer. The steady heavy smokers showed higher cancer incidence and mortality rate than the steady non-smokers (hazard ratio, HR=1.53; 95% CI: 1.49–1.58 and HR=2.64; 95% CI: 2.50–2.79, respectively). The cancer-specific analysis showed that the larynx and lung cancer incidence and mortality rate of the smoking group were higher than in never smokers.

**CONCLUSIONS:**

Smoking, even at low doses, increases the risk of most cancers in men. Quitting or reducing smoking, especially at a young age, can lower cancer incidence and mortality. This study may provide more objective results on the relationship between smoking and cancer, because smoking behavior was examined at multiple time points.

## INTRODUCTION

Smoking behavior can change with time and the changes can result in different health outcomes^1,2^. Cancer risk may change in response to changes in tobacco use and numerous studies have found a link between smoking cessation and cancer-risk reduction^3,4^. Furthermore, the reduction of the risks of specific types of cancers among individuals who decreased the number of cigarettes smoked per day has been reported^5-7^. A systematic review and meta-analysis revealed that substantial smoking reduction may decrease the risk of lung cancer^6^. Smoking reduction or cessation tends to significantly reduce mortality in patients with oral cancer^7^.

In the Republic of Korea (Korea, hereafter), cancer is the leading cause of death and is responsible for 27.5% of all deaths, followed by heart disease, cerebrovascular disease, and pneumonia. The age-standardized cancer incidence rate in 2019 was 295.8 (308.1 and 297.4 among men and women, respectively), and the age-standardized cancer mortality rate in 2020 was 87.9 (124.7 and 60.5 among men and women, respectively) per 100000 persons^8^. For both sexes, stomach, colorectal, and lung cancers, were the most prevalent diagnoses, followed by prostate and liver cancers in men^8^, whereas breast and thyroid cancers were diagnosed more frequently in women. In both men and women, lung cancer was the leading cause of death, followed by liver and stomach cancer in men, and colorectal and stomach cancer in women^9^. Tobacco smoking is a well-known risk factor for cancer, and accounts for 11.8% of cancer incidences and 22.7% of cancer deaths, in Korea^10^. Despite the decrease from 66.3% in 1998 to 36.7% in 2018, the smoking rate among Korean men remains higher than the smoking rate in other developed countries^11^. The relationship between smoking and cancer incidence and mortality in Korea was demonstrated in a previous study^10^. However, there is limited evidence of the long-term health effects of patterns of smoking behavior. In this study, we explored the trajectory of smoking and investigated the associations between the smoking trajectory and cancer incidence and mortality among adult Korean men.

## METHODS

### Data source

This study involved a secondary analysis of data from the National Health Insurance Service (NHIS) for 2002–2018. The NHIS is a mandatory single-payer insurance service that offers benefits for medical services and provides complimentary biennial general health screening for all insured adults. The eligible population has been expanded and, currently, all insured adults are eligible for a biannual general health screening program (annually for manual workers). The participation of the eligible population in the general health screening program has improved from 30% in 2002–2003^12^ to 74% in 2019^12^. Therefore, the NHIS general health screening database contains demographic information, diagnoses, death dates, and information from health examinations, such as data from health questionnaire survey responses, and physical examination and biochemical test results.

### Participants and study design

**Figure 1:**
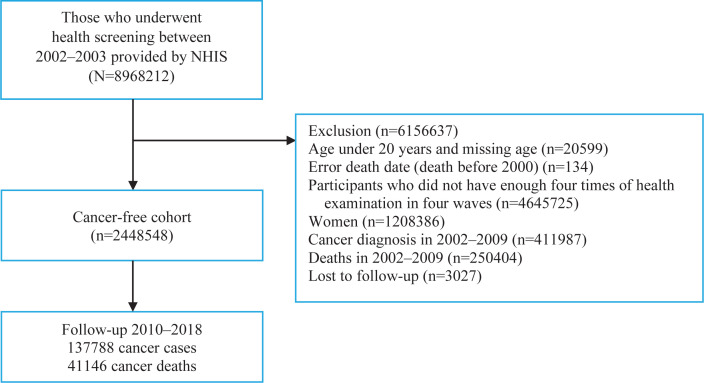
Flow diagram of the selection of study participants

This study used a customized NHIS database of 8968212 people who participated in the 2002–2003 nationwide health examination and were followed up until 2018. Men who underwent four health examinations in 2002–2003, 2004–2005, 2006–2007, and 2008–2009, were included in our analyses. We excluded women because of their very low smoking rate (4–8%)^13^. Furthermore, we excluded 411987 cancer patients and 250404 deaths before 1 January 2010 to generate a cancer-free cohort; additionally, participants younger than 20 years or those with an invalid death date were excluded. A total of 2448548 men were included in the final analysis. The study flow is illustrated in Figure 1.

As this study used anonymized secondary data, the study was exempt from review by the Institutional Review Board of the National Cancer Center, Korea (NCC2018-0279) and the requirement for informed consent was waived.

### Measurements

We assessed smoking behavior in 4 waves between 2002 and 2009. The smoking status was ascertained from the responses to the health questionnaire survey. Based on smoking behaviors, participants were categorized into five groups (non-smokers, former smokers, 1–9 cigarettes per day, 10–19 cigarettes per day, and more than 20 cigarettes per day). Individuals who provided implausible responses as having smoked before in the previous wave and having never smoked in the next wave were recorded as former smokers. Using a group-based trajectory modeling analysis, we divided the study population into 6 smoking trajectory groups: never smokers, former smokers, new current smokers (those who were never smokers at baseline but started smoking later), decreasing light smokers (those who were light smokers but tend to decrease or quit smoking), steady moderate smokers (those who were moderate smokers and did not much change their smoking behavior), and steady heavy smokers (those who were heavy smokers and did not change much their smoking behavior).

Data on covariates, including age, income level (lowest quartile, second quartile, third quartile, and highest quartile), body mass index, alcohol consumption and physical activity (does not do exercise, exercises 1–2 times per week, exercises 3–4 times per week, exercises 5–6 times per week, and exercises almost every day), were collected from the first wave in 2002–2003.

We selected 11 types of cancer that are well-known as being smoking-related cancers^14^. The International Classification of Diseases 10th Revision (ICD-10) codes for individual cancer sites were: oral cancer (C00–C14), esophagus (C15), stomach (C16), colon and rectum (C18–C20), liver (C22), pancreas (C25), larynx (C32), lung cancer (C33–C34), kidney cancer (C64), bladder cancer (C67), and leukemia (C91–C95).

### Statistical analysis

We used group-based trajectory modeling analysis to identify the smoking patterns of 2448548 men, more than 4 times between 2002 and 2009. Group-based modeling methods were used to identify distinct clusters of individuals with similar patterns of outcomes that were measured over time. The PROC TRAJ package in SAS was used for group-based trajectory modeling analysis.

The group-based trajectory modeling analysis revealed 2 criteria for selecting the model: the number of groups and the polynomial of each group. To identify the best-fitted model, we ran all the models using 1 to 6 for the number of groups and 0 to 3 for the polynomial. Bayesian Information Criterion (BIC) was used to evaluate the model fit, wherein a lower BIC indicated a better model fit. Furthermore, we referred to previous studies that analyzed smoking trajectory^15^ to choose an appropriate model. Finally, the model with 6 groups and the groups with polynomials of each group as 0, 3, 3, 3, 3, and 3, were selected as the best-fitting models.

The association between smoking trajectory and specific-site cancer incidence and mortality was analyzed using the Cox proportional hazard model. Censored cases included deaths or having no event of interest during the follow-up period (1 January 2010 to 31 December 2018). The time-to-event (year) was defined as the duration from the beginning of the follow-up to the date of cancer diagnosis, censoring, or the end of follow-up, whichever occurred first. Age, income level, body mass index, alcohol consumption, and physical activity, were used for model adjustment. Chronic viral hepatitis B and C infection (ICD10: B18) were adjusted for the analysis of liver cancer. Subgroup analysis of the Cox proportional hazard model was conducted by age group. Statistical analysis was performed using SAS 9.4 and R software.

## RESULTS

Table 1 shows the general characteristics of the study population, and the mean age was 40.90 years (SD=11.21), with the majority of participants in the age group of 20–59 years (93.19%); only 6.82% were older than 60 years. The mean BMI (kg/m^2^) was 23.86 (SD=2.88). The proportions of non-drinking and light-drinking participants were 28.37% and 31.74%, respectively. Nearly half of the participants were physically inactive. The prevalence of chronic viral hepatitis B or C infection was 11.27%.

**Table 1:** General characteristics of the participants at baseline

*Characteristics*	*n*	*%*
**Age**, mean ± SD	40.90 ± 11.21	
**Age** (years)		
20–29	390484	15.95
30–39	817602	33.39
40–49	727021	29.69
50–59	346465	14.15
≥60	166976	6.82
**BMI** (kg/m^2^), mean ± SD	23.86 ± 2.88	
**Income level quartile**		
Lowest	261121	11.14
Second	514727	21.96
Third	733098	31.28
Highest	834788	35.62
**Smoking**		
Never smokers	893146	36.48
Former smokers	393267	16.06
1–9 cigarettes/day	261754	10.69
10–19 cigarettes/day	681585	27.84
≥20 cigarettes/day	218796	8.93
**Alcohol consumption** (g/day)		
0	693084	28.37
1–9	775266	31.74
10–19	640039	26.20
20–29	192257	7.87
30–49	53698	2.20
≥50	88527	3.62
**Physical activity** (times per week)		
0	1063309	44.11
1–2	882517	36.61
3–4	289241	12.00
5–6	62642	2.60
Almost everyday	112621	4.67
**Chronic viral hepatitis B or C**		
No	2172585	88.73
Yes	275963	11.27

**Figure 2:**
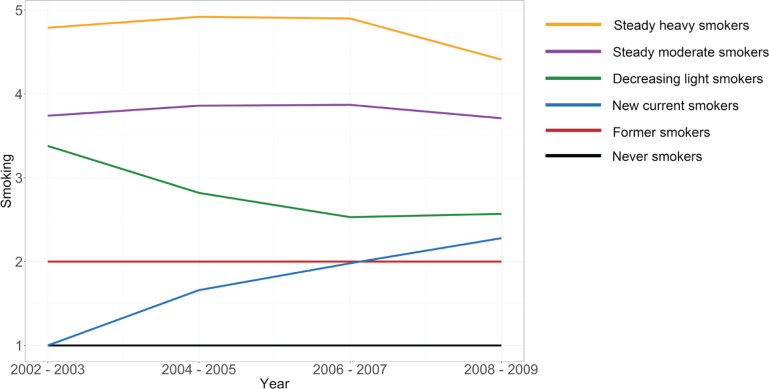
Smoking trajectory groups

Figure 2 shows the smoking trajectory groups. Based on the lowest BIC, we derived six smoking trajectory groups as follows: Group 1 (22.05%, never smokers), Group 2 (11.16%, former smokers), Group 3 (12.75%, new current smokers), Group 4 (20.22%, decreasing light smokers), Group 5 (29.52%, steady moderate smokers), and Group 6 (4.30%, steady heavy smokers).

Table 2 shows the baseline characteristics of participants stratified by their smoking trajectory. New current smokers had the highest mean age (44.63 ± 12.09 years), followed by former smokers (43.96 ± 10.67), and never smokers (42.37 ± 11.97). Participants who were older than 60 years accounted for the highest proportions of new current smokers; only 1.12% of participants older than 60 years were steady heavy smokers. The steady moderate smokers and steady heavy smokers had lower incomes than those in the other groups. The steady moderate smokers and steady heavy smokers tended to drink more alcohol than other groups; 4.38% of the steady moderate smokers and 9.72% of the steady heavy smokers drank more than 50 g of ethanol per day.

**Table 2:** General characteristic of study participants at baseline by smoking trajectory group (2002–2003)

*Characteristics*	*Never smokers n (%)*	*Former smokers n (%)*	*New current smokers n (%)*	*Decreasing light smokers n (%)*	*Steady moderate smokers n (%)*	*Steady heavy smokers n (%)*
**Age** (years), mean ± SD	42.37 ± 11.97	43.96 ± 10.67	44.63 ± 12.09	40.22 ± 10.97	37.94 ± 9.90	37.99 ± 8.53
**Age** (years)						
20–29	80391 (14.89)	19237 (7.04)	30891 (9.89)	84056 (16.97)	158408 (21.91)	17501 (16.62)
30–39	160294 (29.70)	78510 (28.74)	84792 (27.16)	174725 (35.28)	274891 (38.03)	44390 (42.15)
40–49	156063 (28.91)	102632 (37.57)	96364 (30.87)	143046 (28.89)	195824 (27.09)	33092 (31.42)
50–59	91760 (17.00)	49244 (18.03)	61070 (19.56)	62725 (12.67)	72538 (10.03)	9128 (8.67)
≥60	51290 (9.50)	23525 (8.61)	39081 (12.52)	30632 (6.18)	21249 (2.94)	1199 (1.14)
**BMI** (kg/m^2^)						
Underweight	9809 (1.82)	3285 (1.20)	5225 (1.68)	11192 (2.26)	18732 (2.59)	1815 (1.72)
Normal weight	191024 (35.42)	85637 (31.37)	105013 (33.67)	187098 (37.82)	285577 (39.54)	34951 (33.21)
Overweight	154652 (28.68)	82349 (30.17)	90225 (28.93)	135763 (27.44)	186187 (25.78)	26826 (25.49)
Obese	183760 (34.08)	101685 (37.25)	111410 (35.72)	160638 (32.47)	231743 (32.09)	41639 (39.57)
**Income level quartile**						
Lowest	54617 (10.56)	19996 (7.59)	33279 (11.01)	51051 (10.80)	89175 (12.98)	13003 (12.87)
Second	103891 (20.08)	43028 (16.32)	60512 (20.03)	103239 (21.84)	178018 (25.92)	26039 (25.76)
Third	157269 (30.40)	79023 (29.98)	90660 (30.01)	149872 (31.71)	222637 (32.41)	33637 (33.28)
Highest	201577 (38.96)	121570 (46.12)	117685 (38.95)	168480 (35.65)	197084 (28.69)	28392 (28.09)
**Smoking** (cigarettes/day)						
Never smokers	539798 (100)	0 (0.00)	312198 (100)	2372 (0.48)	38778 (5.36)	0 (0.00)
Former smokers	0 (0.00)	273148 (100)	0 (0.00)	81808 (16.52)	38311 (5.30)	0 (0.00)
1–9	0 (0.00)	0 (0.00)	0 (0.00)	176135 (35.57)	85619 (11.84)	0 (0.00)
10–19	0 (0.00)	0 (0.00)	0 (0.00)	203627 (41.12)	454114 (62.82)	23844 (22.64)
≥20	0 (0.00)	0 (0.00)	0 (0.00)	31242 (6.31)	106088 (14.68)	81466 (77.36)
**Alcohol consumption** (g/day)						
0	226489 (42.12)	62119 (22.83)	138694 (44.77)	94766 (19.22)	150167 (20.84)	19390 (18.45)
1–9	175030 (32.55)	95087 (34.94)	82518 (26.63)	178839 (36.27)	219301 (30.44)	23135 (22.01)
10–19	95806 (17.82)	75331 (27.68)	57059 (18.42)	149847 (30.39)	229104 (31.80)	31809 (30.26)
20–29	24189 (4.50)	23397 (8.60)	18682 (6.03)	41281 (8.37)	69768 (9.68)	14610 (13.90)
30–49	5656 (1.05)	6096 (2.24)	4117 (1.33)	11174 (2.27)	20605 (2.86)	5958 (5.67)
≥50	10561 (1.96)	10097 (3.71)	8745 (2.82)	17195 (3.49)	31539 (4.38)	10216 (9.72)
**Physical activity** (times per week)						
0	227714 (42.85)	98434 (36.55)	144501 (47.19)	212561 (43.64)	325835 (45.81)	54264 (51.69)
1–2	191440 (36.02)	101524 (37.70)	96355 (31.47)	184978 (37.97)	272543 (38.32)	35677 (33.98)
3–4	67211 (12.65)	43469 (16.14)	38866 (12.69)	57064 (11.71)	73152 (10.28)	9479 (9.03)
5–6	14958 (2.81)	9914 (3.68)	9051 (2.96)	11720 (2.41)	14976 (2.11)	2023 (1.93)
Almost everyday	30148 (5.67)	15971 (5.93)	17411 (5.69)	20786 (4.27)	24769 (3.48)	3536 (3.37)
**Chronic viral hepatitis**						
No	474808 (87.96)	239570 (87.71)	273225 (87.52)	439903 (88.84)	650432 (89.97)	94647 (89.87)
Yes	64990 (12.04)	33578 (12.29)	38973 (12.48)	55281 (11.16)	72478 (10.03)	10663 (10.13)

Cancer incidence was associated with smoking intensity over time (Supplementary file Figure 2). Groups other than never smokers had a higher risk of developing cancer for almost all types of cancer. The steady moderate smokers and steady heavy smokers showed the highest risk of cancer. For all cancer sites, the adjusted hazard ratio was 1.10 (95% CI: 1.08–1.13) for former smokers, 1.13 (95% CI: 1.11–1.15) for new current smokers, 1.30 (95% CI: 1.28–1.33) for the decreasing light smokers, 1.45 (95% CI: 1.43–1.48) for the steady moderate smokers, and 1.53 (95% CI: 1.49–1.58) for steady heavy smokers. Of the 11 types of cancer, the risks of developing laryngeal and lung cancers were the highest in the smoking group. The risks of lung and laryngeal cancers in the steady heavy smokers were 7.45 (95% CI: 6.88–8.08) and 9.57 (95% CI: 7.03–13.01), respectively.

The pattern of cancer mortality was relatively similar to that of cancer incidence (Supplementary file Figure 3). A higher risk of cancer mortality was seen in all the groups other than never smokers group for almost all types of cancer. Steady moderate smokers and steady heavy smokers often showed the highest risk of cancer mortality. With regard to the cancer sites, the adjusted hazard ratio was 1.17 (95% CI: 1.13–1.21) for former smokers, 1.22 (95% CI: 1.17–1.26) for new current smokers, 1.68 (95% CI: 1.63–1.73) for the decreasing light smokers, 2.22 (95% CI: 2.15–2.29) for the steady moderate smokers, and 2.64 (95% CI: 2.50–2.79) for the steady heavy smokers. Of the 11 types of cancer, the mortality risks from lung and laryngeal cancer in the smoking group were highest and were 9.94 (95% CI: 9.02–10.95) and 11.51 (95% CI: 5.43–24.39) in steady heavy smokers, respectively.

The results of the subgroup analysis of smoking trajectory and cancer incidence by age group are shown in Supplementary file Table 1. There was no risk of developing cancer in the youngest age group (20–29 years). The cancer risk in the 40–49 years and 50–59 years groups was often higher than that of their younger counterparts, especially for lung and laryngeal cancer. Although the decreasing light smokers attempted to decrease or quit smoking, their cancer risk remained high. However, participants in the young adult group showed a lower cancer risk than those in the middle-aged or older adult group. A similar pattern was found for the smoking trajectory and cancer mortality by age group (Supplementary file Table 2). The mortality risk was not detected in people aged 20–29 years. The cancer mortality risks in the 50–59 years and >60 years groups were highest for lung and laryngeal cancers.

## DISCUSSION

In this study, the trajectory analysis for smoking behavior was measured 4 times from 2002 to 2009 and helped to identify six groups of smoking behaviors. Steady moderate smokers appeared to be the most common group, accounting for 29.52% of the participants, followed by never smokers (22.05%), decreasing light smokers (20.22%), and new current smokers (12.75%). Meanwhile, the proportion of former smokers and steady heavy smokers were lowest, at 11.16% and 4.30%, respectively. Several previous studies have investigated the smoking trajectory and the number of trajectories varied from 3 to 6^15-20^. Analysis of the smoking trajectory from two previous studies conducted by Jee et al.^15,20^ in Korea also revealed five and six smoking groups. Nevertheless, the smoking patterns and the proportion of each group are different from those of our study, which can be explained by the dissimilarities of the number of data points, smoking indicators used, and time axes^21^. Whereas Jee et al.^15,18^ utilized seven repeated surveys every 2 years from 1992 to 2005 in both studies, the number of data repetitions in our study amounted to only 4 times between 2002 and 2009. In addition, the difference originated from the participants. The sample in our study was larger, and we included all adult men aged ≥20 years for the analysis, whereas the studies of Jee et al.^15,18^ only recruited young individuals aged 20–39 years or 20–29 years.

The relationship between smoking and cancer risk has been well-established; however, to the best of our knowledge, this is the first study to assess how smoking trajectories influence the risk of all cancers. Our analysis indicates that adult Korean men in all identified groups, namely former smokers, new current smokers, decreasing light smokers, steady moderate smokers, and steady heavy smokers, had significantly increased risks of morbidity and mortality from all cancers combined. According to the adjusted results for potential confounding variables, all smoking groups were at higher risk of cancer and mortality than never smokers, of which steady moderate smokers and steady heavy smokers were the two most at-risk groups. The association between smoking and cancer risk, especially in moderate and heavy smokers, was also supported by a meta-analysis of 254 studies^22^. On the other hand, in this study, the remaining smoking trajectory groups, including former smokers, new current smokers, and decreasing light smokers, had lower risks than the above two groups but higher risks than never smokers. The risk of cancer of new current smokers in our study, albeit lower than those of the other smoking groups, was significantly increased relative to that of participants who had never smoked. A large cohort study in the US conducted in 2018 showed no significant association between incident smoking-related cancer risk and short-duration smoking (<10 years)^23^. However, the abovementioned study only focused on the low intensity of smoking; in our study, new current smokers included moderate and heavy smokers (Supplementary file Figure 1B). Several studies confirmed the significant risk of many smoking-related diseases, namely cancer, cardiovascular disease, and all-cause mortality, associated with low-intensity daily smoking^24-26^. A population-based cohort study in Australia found an increase in the risks of all cancers in current smokers, as well as ‘light’ smokers^27^. Two cohort studies in the US indicated that smokers of fewer than ten cigarettes per day were at increased risk of cancer relative to never smokers^23,28^. Despite the proven effect of smoking cessation and smoking reduction on cancer risk, people who quit smoking still face a higher risk than people who have never smoked. A study in Korea by Choi et al.^3^ showed that heavy or moderate smoking quitters, compared with non-smokers, had a higher risk of cancer. Furthermore, men who reduced the number of cigarettes per day had reduced risks of all cancers and the highest risk among heavy continual smokers^3^. However, another study of Korean males conducted by Song et al.^29^ showed that reduced smoking was negatively associated with only some specific types of cancers rather than all cancers. The divergent research results may be attributable to the different ages of the study participants. Age has been recognized as a critical risk factor for cancer and many individual cancer types^30^. The participants of the studies by Song et al.^29^ and Choi et al.^3^ were in the age groups of 30–58 and 40–82 years, respectively, while our study had no age restriction and included young males in their 20s to older men (>60 years). In addition, different smoking status assessments can cause inconsistent results. In the study by Song et al.^29^, the change in smoking status was tracked between 1990 and 1992, while this change was looked at for a longer period and measured repeated 4 times in our study. Furthermore, differing smoking status measures can lead to inconsistencies in results. The change in smoking status was monitored in the study by Song et al.^29^ between 1990 and 1992. However, this change was examined over a long period in our study and repeated 4 times.

Further investigations showed increased smoking-associated risks of cancers of the lip, oral cavity, and pharynx, esophagus, stomach, colorectum, liver, pancreas, larynx, lung, and bladder, and death. Meanwhile, the current study demonstrated no significant association between smoking and kidney cancer incidence and mortality, which was consistent with previous studies conducted in Australia, China, and Japan^27,31,32^. For the Korean population, a meta-analysis in 2014 revealed similar results on the types of cancers with increased risk, except for colorectal cancer10. In our study, the relationship between colorectal cancer and smoking was statistically significant; however, this relationship was not shown in the previous meta-analysis^10^.

Our study consolidated previous findings that smoking was associated with the highest risks of lung cancer and laryngeal cancer^10,27,28,32^. However, the magnitude of the risk of smoking groups compared to never smokers varies from study to study. A 7.45-fold higher risk of lung cancer was observed in the steady heavy smokers in the current study, contributing to 9.94 times higher lung cancer mortality than never smokers. The risk magnitudes of cancer incidence and mortality among smokers in our study were comparable with those reported by studies in Asia but lower than those reported by other cohort studies in Western countries. The relative risk of lung cancer associated with smoking among men was 5.70 (95% CI: 3.00–7.50) in a study in China and 3.85 (95% CI: 3.12–4.74) in Japan^31,32^. A population-based Australian cohort study showed that hazards escalated with increasing smoking intensity; for lung cancer, the hazard ratio was 9.22 (95% CI: 5.14–16.55) for 1–5 cigarettes/day and 38.61 (95% CI: 25.65–58.13) for >35 cigarettes/day^27^. A study from the US revealed hazard ratios of 17.3 (95% CI: 14.3–20.9) for participants smoking 1–10 cigarettes/day and 53 (95% CI: 43.2–65.2) for >40 cigarettes/day for lung cancer^28^. The difference in hazard ratio recorded in these studies and our study can be due to several factors, such as the assessment of smoking, participant characteristics, and participant follow-up duration. For example, in studies in Australia and the US, smoking status was collected only once during the baseline, as opposed to our study, where it was tracked for eight years and grouped based on smoking characteristics^27,28^. On the other hand, some demographic characteristics, such as race and continent of residence, have been reported to contribute to the risk of smoking-related cancers^22,33,34^. For instance, white individuals seem more susceptible to the impact of tobacco smoke than Asians^34^. Genetic differences might explain differences in cancer risk between smokers who consume the same amount of cigarettes^35^. Results from a meta-analysis showed that cancer risk was slightly higher in the Western than Asian countries^22^. In addition, gaps in the follow-up periods can have a large effect on the latent period for cancer development. Our study and other Asian studies had a relatively short duration of follow-up relative to studies conducted in western countries.

For subgroup analysis, we divided the participants into five age groups: 20–29, 30–39, 40–49, 50–59, and ≥60 years. The risk of all cancers and the combined mortality rate increased with age. Among the young adults aged 20–29 years, the cancer incidence and mortality risk of former smokers, new current smokers, decreasing light smokers, steady moderate smokers, and steady heavy smokers, were not significant. In addition, while cutting down and quitting smoking appeared to be effective in reducing the risk of cancers and cancer-related mortality for young adults, this was not observed in middle-aged and older adults. For lung cancer, the risk was significantly lower in the younger decreasing light smokers than in the older group. However, differences in cancer risk by age group should be carefully considered due to the insufficient follow-up to determine smoking behavior and incidence of cancers. Data on smoking behaviors before this study were inaccessible, and they were not included in the current analysis.

### Strengths and limitations

Results of this study should be used in light of the following limitations. First, misclassification of smoking status may have occurred due to the nature of self-reported data. Second, the research period of this study may have been insufficient to provide concrete evidence on lifetime changes in smoking and their long-term effect on cancer. Third, only people who participated in health examinations four consecutive times were selected for this study, and the results should be generalized cautiously. Despite these shortcomings, this research made a notable contribution to the current literature. A strength of our study is that the changes in smoking patterns (e.g. smoking intensity, initiation into smoking, etc.) were monitored over the research period and taken into account in relation to the outcomes of interest. We also used data from a population-based cohort study, which covered a large number of residents and a long follow-up duration.

## CONCLUSIONS

Previous studies on the association between smoking and cancer produced results that were broadly equivalent to those obtained in our study. However, there were some differences in the associations between smoking and some specific types of cancer; besides, the magnitudes of these associations varied between the studies. Smoking was followed across time rather than at a single point, and the association between smoking trajectory and cancer in this study may provide more objective evidence. Cancer incidence and mortality rates increased with the number of cigarettes smoked per day and the smoking duration. Even with a low smoking intensity or within a short time, smoking increases the risk of most cancers in adult males. People who quit or reduce their smoking, especially at young ages, can have lower cancer incidence and mortality risks.

## Data Availability

The data supporting this research cannot be made available for privacy or other reasons. The data used in this study (NHIS-2020-1-396) was provided by the National Health Insurance Service. It is available for researchers who meet the criteria for access to confidential data. For the protection of personal information, the data cannot be shared because NHIS prohibits the transfer, rental, or sale of the database to third parties except for researchers who have been approved for access. The NHIS data can be requested through its website (https://nhiss.nhis.or.kr).
