# Supplementary material for: Smoking trajectory and cancer risk: A population-based cohort study
Source: Tob Induc Dis. 2022 Aug 26;20:71. doi: 10.18332/tid/152137 (PMC9413414; doi:10.18332/tid/152137)
Supplement: Supplementary file 1 [file TID-20-71-s1.pdf]

Appendix Table 1. Smoking trajectory group and cancer incidence by age group

Cancer site	Adjusted hazard ratio (95% CI)				
	20-29 years	30-39 years	40-49 years	50-59 years	≥60 years
All cancer					
Never smokers	ref	ref	ref	ref	ref
Former smokers	0.94 (0.82 - 1.08)	0.97 (0.91 - 1.03)	1.07 (1.03 - 1.11)	1.11 (1.07 - 1.15)	1.2 (1.16 - 1.25)
New current smokers	1.03 (0.92 - 1.16)	1.06 (1.00 - 1.12)	1.11 (1.07 - 1.15)	1.13 (1.10 - 1.17)	1.21 (1.17 - 1.25)
Decreasing light smokers	1.01 (0.93 - 1.10)	1.08 (1.03 - 1.13)	1.24 (1.20 - 1.28)	1.33 (1.29 - 1.37)	1.45 (1.41 - 1.50)
Steady moderate smokers	0.94 (0.87 - 1.01)	1.12 (1.07 - 1.17)	1.40 (1.36 - 1.44)	1.57 (1.52 - 1.61)	1.71 (1.65 - 1.77)
Steady heavy smokers	0.88 (0.76 - 1.02)	1.13 (1.06 - 1.22)	1.50 (1.43 - 1.57)	1.81 (1.71 - 1.91)	2.05 (1.84 - 2.29)
Lip, oral cavity and pharynx					
Never smokers	ref	ref	ref	ref	ref
Former smokers	0.50 (0.11 - 2.17)	1.13 (0.70 - 1.83)	1.00 (0.74 - 1.35)	1.35 (0.98 - 1.85)	1.19 (0.82 - 1.72)
New current smokers	1.40 (0.59 - 3.30)	1.24 (0.78 - 1.97)	1.01 (0.74 - 1.37)	1.42 (1.06 - 1.92)	1.25 (0.91 - 1.72)
Decreasing light smokers	1.29 (0.66 - 2.50)	1.27 (0.86 - 1.87)	1.10 (0.84 - 1.44)	1.58 (1.18 - 2.10)	1.69 (1.23 - 2.32)
Steady moderate smokers	1.21 (0.66 - 2.23)	1.60 (1.13 - 2.27)	1.61 (1.27 - 2.04)	2.23 (1.71 - 2.90)	2.10 (1.51 - 2.94)
Steady heavy smokers	0.87 (0.25 - 3.05)	2.36 (1.48 - 3.77)	1.15 (0.76 - 1.76)	3.07 (2.00 - 4.7)	2.67 (1.08 - 6.62)
Esophagus					
Never smokers	ref	ref	ref	ref	ref
Former smokers	n/a	3.37 (0.84 - 13.50)	1.40 (0.91 - 2.16)	1.75 (1.28 - 2.38)	1.35 (1.02 - 1.78)
New current smokers	n/a	2.86 (0.68 - 11.97)	1.62 (1.05 - 2.50)	1.55 (1.15 - 2.11)	1.16 (0.90 - 1.50)
Decreasing light smokers	0.81 (0.05 - 13.04)	3.24 (0.91 - 11.52)	2.08 (1.43 - 3.03)	2.34 (1.78 - 3.08)	1.53 (1.19 - 1.96)
Steady moderate smokers	3.02 (0.36 - 24.99)	6.53 (2.01 - 21.20)	3.66 (2.60 - 5.14)	3.11 (2.41 - 4.02)	1.68 (1.29 - 2.19)
Steady heavy smokers	3.34 (0.20 - 56.97)	9.57 (2.65 - 34.65)	3.49 (2.24 - 5.45)	3.23 (2.16 - 4.84)	1.55 (0.68 - 3.53)
Stomach					
Never smokers	ref	ref	ref	ref	ref
Former smokers	0.99 (0.64 - 1.53)	1.20 (1.04 - 1.38)	1.20 (1.11 - 1.31)	1.18 (1.09 - 1.28)	1.28 (1.17 - 1.40)
New current smokers	1.30 (0.92 - 1.82)	1.32 (1.15 - 1.51)	1.28 (1.18 - 1.39)	1.15 (1.07 - 1.24)	1.32 (1.22 - 1.42)
Decreasing light smokers	1.23 (0.95 - 1.60)	1.39 (1.24 - 1.56)	1.48 (1.38 - 1.59)	1.45 (1.36 - 1.56)	1.50 (1.38 - 1.62)
Steady moderate smokers	1.58 (1.26 - 1.99)	1.62 (1.46 - 1.80)	1.75 (1.64 - 1.87)	1.60 (1.50 - 1.71)	1.56 (1.43 - 1.71)
Steady heavy smokers	1.42 (0.95 - 2.13)	1.87 (1.61 - 2.18)	1.80 (1.62 - 2.00)	1.51 (1.31 - 1.73)	1.61 (1.20 - 2.16)
Colorectum					
Never smokers	ref	ref	ref	ref	ref
Former smokers	0.70 (0.42 - 1.17)	1.02 (0.87 - 1.20)	1.18 (1.07 - 1.30)	1.10 (1.01 - 1.21)	1.19 (1.08 - 1.32)
New current smokers	1.31 (0.93 - 1.83)	1.09 (0.93 - 1.27)	1.14 (1.04 - 1.26)	1.13 (1.03 - 1.22)	1.16 (1.06 - 1.26)
Decreasing light smokers	1.12 (0.85 - 1.46)	1.05 (0.92 - 1.20)	1.29 (1.19 - 1.41)	1.39 (1.28 - 1.50)	1.26 (1.15 - 1.38)
Steady moderate smokers	0.98 (0.77 - 1.26)	1.17 (1.04 - 1.31)	1.42 (1.31 - 1.54)	1.35 (1.25 - 1.46)	1.24 (1.12 - 1.38)
Steady heavy smokers	1.16 (0.75 - 1.78)	1.03 (0.85 - 1.26)	1.39 (1.22 - 1.58)	1.26 (1.07 - 1.50)	1.18 (0.82 - 1.71)
Liver					

Never smokers	ref	ref	ref	ref	ref
Former smokers	0.78 (0.34 - 1.76)	0.77 (0.60 - 0.98)	1.07 (0.94 - 1.21)	1.02 (0.90 - 1.16)	1.02 (0.90 - 1.16)
New current smokers	1.49 (0.85 - 2.59)	1.21 (0.99 - 1.49)	1.31 (1.17 - 1.48)	1.16 (1.03 - 1.29)	1.16 (1.03 - 1.29)
Decreasing light smokers	1.52 (0.98 - 2.35)	1.39 (1.17 - 1.66)	1.76 (1.59 - 1.95)	1.35 (1.21 - 1.51)	1.35 (1.21 - 1.51)
Steady moderate smokers	1.29 (0.85 - 1.95)	1.78 (1.52 - 2.08)	1.93 (1.75 - 2.13)	1.54 (1.39 - 1.71)	1.54 (1.39 - 1.71)
Steady heavy smokers	1.56 (0.80 - 3.07)	0.95 (0.71 - 1.27)	1.47 (1.24 - 1.75)	1.27 (1.01 - 1.60)	1.27 (1.01 - 1.60)
Pancreas					
Never smokers	ref	ref	ref	ref	ref
Former smokers	1.97 (0.59 - 6.55)	0.98 (0.62 - 1.55)	0.95 (0.74 - 1.21)	1.05 (0.86 - 1.30)	1.18 (0.96 - 1.45)
New current smokers	2.08 (0.75 - 5.75)	1.04 (0.66 - 1.62)	1.01 (0.79 - 1.29)	1.12 (0.92 - 1.35)	1.08 (0.90 - 1.29)
Decreasing light smokers	1.04 (0.40 - 2.70)	1.16 (0.80 - 1.67)	1.24 (1.00 - 1.53)	1.26 (1.04 - 1.52)	1.23 (1.01 - 1.49)
Steady moderate smokers	1.29 (0.57 - 2.95)	1.56 (1.13 - 2.15)	1.54 (1.27 - 1.87)	1.50 (1.26 - 1.79)	1.83 (1.50 - 2.23)
Steady heavy smokers	0.47 (0.06 - 3.78)	1.98 (1.25 - 3.12)	1.83 (1.36 - 2.46)	2.15 (1.57 - 2.95)	1.53 (0.76 - 3.11)
Larynx					
Never smokers	ref	ref	ref	ref	ref
Former smokers	4.34 (0.27 - 70.43)	1.25 (0.35 - 4.43)	1.91 (1.05 - 3.47)	3.10 (1.80 - 5.32)	1.66 (1.03 - 2.68)
New current smokers	n/a	2.67 (0.95 - 7.49)	2.08 (1.14 - 3.78)	2.56 (1.50 - 4.39)	1.58 (1.03 - 2.43)
Decreasing light smokers	1.93 (0.17 - 21.62)	1.80 (0.67 - 4.80)	3.45 (2.07 - 5.78)	4.45 (2.72 - 7.28)	2.85 (1.91 - 4.25)
Steady moderate smokers	1.48 (0.15 - 14.53)	2.55 (1.05 - 6.21)	5.88 (3.64 - 9.52)	9.24 (5.84 - 14.63)	3.58 (2.37 - 5.42)
Steady heavy smokers	5.03 (0.29 - 85.87)	4.36 (1.52 - 12.51)	8.47 (4.85 - 14.82)	15.03 (8.63 - 26.19)	6.29 (2.64 - 14.98)
Lung					
Never smokers	ref	ref	ref	ref	ref
Former smokers	1.16 (0.53 - 2.56)	1.17 (0.89 - 1.53)	1.20 (1.04 - 1.40)	1.47 (1.29 - 1.67)	2.26 (2.02 - 2.52)
New current smokers	1.34 (0.71 - 2.52)	0.92 (0.69 - 1.23)	1.23 (1.06 - 1.44)	1.64 (1.46 - 1.84)	2.16 (1.96 - 2.39)
Decreasing light smokers	1.14 (0.68 - 1.90)	1.10 (0.88 - 1.38)	1.66 (1.46 - 1.89)	2.88 (2.60 - 3.20)	4.36 (3.97 - 4.79)
Steady moderate smokers	1.28 (0.81 - 2.01)	1.52 (1.24 - 1.85)	2.85 (2.54 - 3.19)	5.23 (4.75 - 5.75)	6.75 (6.14 - 7.41)
Steady heavy smokers	1.63 (0.78 - 3.41)	2.20 (1.66 - 2.91)	4.66 (4.03 - 5.40)	8.46 (7.43 - 9.64)	10.09 (8.35 - 12.19)
Kidney					
Never smokers	ref	ref	ref	ref	ref
Former smokers	0.59 (0.27 - 1.31)	0.89 (0.69 - 1.16)	0.96 (0.80 - 1.16)	1.07 (0.87 - 1.32)	0.89 (0.66 - 1.21)
New current smokers	0.82 (0.46 - 1.46)	1.07 (0.84 - 1.36)	1.00 (0.83 - 1.20)	1.07 (0.88 - 1.31)	0.97 (0.75 - 1.25)
Decreasing light smokers	1.06 (0.71 - 1.58)	1.02 (0.84 - 1.25)	1.04 (0.88 - 1.23)	0.84 (0.68 - 1.05)	1.06 (0.81 - 1.40)
Steady moderate smokers	1.25 (0.89 - 1.77)	0.97 (0.81 - 1.17)	1.07 (0.91 - 1.25)	1.11 (0.92 - 1.35)	1.04 (0.75 - 1.43)
Steady heavy smokers	0.87 (0.44 - 1.74)	1.02 (0.75 - 1.38)	1.21 (0.93 - 1.58)	1.30 (0.88 - 1.94)	1.72 (0.70 - 4.21)
Bladder					
Never smokers	ref	ref	ref	ref	ref
Former smokers	0.36 (0.05 - 2.79)	1.44 (0.87 - 2.37)	1.51 (1.19 - 1.91)	1.4 (1.16 - 1.70)	1.33 (1.12 - 1.58)
New current smokers	0.47 (0.10 - 2.11)	1.73 (1.08 - 2.76)	1.75 (1.39 - 2.21)	1.46 (1.22 - 1.74)	1.44 (1.25 - 1.67)
Decreasing light smokers	2.02 (0.98 - 4.17)	2.33 (1.58 - 3.43)	1.83 (1.48 - 2.28)	1.81 (1.53 - 2.15)	1.58 (1.36 - 1.85)

Steady moderate smokers	1.50 (0.75 - 3.01)	2.74 (1.91 - 3.95)	2.27 (1.86 - 2.77)	2.26 (1.92 - 2.65)	2.01 (1.71 - 2.37)
Steady heavy smokers	1.70 (0.53 - 5.44)	3.50 (2.18 - 5.62)	2.84 (2.13 - 3.79)	2.69 (2.00 - 3.60)	2.47 (1.51 - 4.02)
Leukemia					
Never smokers	ref	ref	ref	ref	ref
Former smokers	0.94 (0.46 - 1.95)	0.87 (0.58 - 1.31)	1.11 (0.81 - 1.51)	1.16 (0.81 - 1.65)	0.97 (0.69 - 1.36)
New current smokers	1.27 (0.74 - 2.18)	1.24 (0.87 - 1.76)	1.27 (0.94 - 1.72)	1.55 (1.15 - 2.10)	1.22 (0.92 - 1.60)
Decreasing light smokers	0.83 (0.52 - 1.32)	0.92 (0.67 - 1.27)	1.28 (0.97 - 1.69)	1.49 (1.09 - 2.02)	1.30 (0.96 - 1.75)
Steady moderate smokers	0.76 (0.50 - 1.14)	0.92 (0.69 - 1.24)	1.14 (0.87 - 1.49)	1.79 (1.34 - 2.40)	1.54 (1.11 - 2.14)
Steady heavy smokers	0.6 (0.24 - 1.54)	1.09 (0.67 - 1.76)	1.52 (1.00 - 2.31)	1.74 (0.96 - 3.15)	0.48 (0.07 - 3.44)
Others					
Never smokers	ref	ref	ref	ref	ref
Former smokers	0.94 (0.46 - 1.95)	0.90 (0.83 - 0.99)	0.94 (0.89 - 1.00)	0.98 (0.93 - 1.04)	0.99 (0.93 - 1.05)
New current smokers	1.27 (0.74 - 2.18)	0.93 (0.86 - 1.01)	0.92 (0.86 - 0.98)	0.98 (0.93 - 1.03)	1.02 (0.96 - 1.07)
Decreasing light smokers	0.83 (0.52 - 1.32)	0.95 (0.88 - 1.01)	0.92 (0.87 - 0.97)	0.95 (0.90 - 1.01)	0.95 (0.90 - 1.01)
Steady moderate smokers	0.76 (0.50 - 1.14)	0.82 (0.76 - 0.87)	0.84 (0.80 - 0.89)	0.91 (0.86 - 0.96)	0.95 (0.89 - 1.02)
Steady heavy smokers	0.60 (0.24 - 1.54)	0.77 (0.69 - 0.87)	0.86 (0.77 - 0.95)	1.01 (0.90 - 1.14)	1.12 (0.89 - 1.41)

Appendix Table 2. Smoking trajectory group and cancer mortality by age group

Cancer site	Adjusted hazard ratio (95% CI)				
	20-29 years	30-39 years	40-49 years	50-59 years	≥60 years
All cancer					
Never smokers	ref	ref	ref	ref	ref
Former smokers	0.79 (0.48 - 1.29)	0.86 (0.72 - 1.02)	1.07 (0.98 - 1.17)	1.14 (1.06 - 1.23)	1.30 (1.23 - 1.37)
New current smokers	1.34 (0.97 - 1.85)	1.06 (0.91 - 1.24)	1.10 (1.01 - 1.20)	1.21 (1.14 - 1.30)	1.30 (1.24 - 1.36)
Decreasing light smokers	1.06 (0.81 - 1.40)	1.08 (0.94 - 1.22)	1.44 (1.34 - 1.56)	1.69 (1.59 - 1.80)	1.85 (1.77 - 1.94)
Steady moderate smokers	0.95 (0.75 - 1.22)	1.29 (1.15 - 1.44)	1.79 (1.68 - 1.92)	2.28 (2.16 - 2.41)	2.39 (2.28 - 2.51)
Steady heavy smokers	1.31 (0.90 - 1.92)	1.25 (1.04 - 1.49)	2.18 (1.98 - 2.40)	2.80 (2.54 - 3.07)	3.01 (2.62 - 3.46)
Lip, oral cavity and pharynx					
Never smokers	ref	ref	ref	ref	ref
Former smokers	n/a	1.40 (0.31 - 6.26)	0.81 (0.38 - 1.77)	1.57 (0.82 - 3.01)	1.17 (0.72 - 1.92)
New current smokers	5.12 (0.46 - 56.83)	2.16 (0.58 - 8.04)	1.14 (0.56 - 2.33)	2.18 (1.24 - 3.82)	0.99 (0.63 - 1.56)
Decreasing light smokers	n/a	2.82 (0.92 - 8.69)	1.25 (0.67 - 2.33)	2.14 (1.22 - 3.73)	1.53 (0.99 - 2.36)
Steady moderate smokers	3.45 (0.42 - 28.54)	2.57 (0.88 - 7.55)	2.64 (1.55 - 4.48)	2.85 (1.69 - 4.80)	2.18 (1.40 - 3.40)
Steady heavy smokers	3.73 (0.22 - 62.70)	3.26 (0.86 - 12.44)	2.73 (1.29 - 5.78)	6.06 (3.05 - 12.03)	3.93 (1.4 - 11.05)
Esophagus					
Never smokers	ref	ref	ref	ref	ref
Former smokers	n/a	1.92 (0.27 - 13.72)	1.08 (0.50 - 2.30)	1.71 (1.03 - 2.85)	1.46 (1.04 - 2.05)
New current smokers	n/a	n/a	1.13 (0.52 - 2.46)	1.42 (0.86 - 2.36)	1.25 (0.92 - 1.71)
Decreasing light smokers	n/a	2.04 (0.39 - 10.54)	2.54 (1.40 - 4.58)	2.74 (1.78 - 4.22)	1.56 (1.15 - 2.12)
Steady moderate smokers	n/a	3.26 (0.74 - 14.28)	3.49 (2.01 - 6.07)	3.44 (2.28 - 5.20)	1.82 (1.31 - 2.51)
Steady heavy smokers	n/a	6.03 (1.18 - 30.72)	3.82 (1.91 - 7.67)	3.81 (2.07 - 7.00)	1.24 (0.39 - 3.94)
Stomach					
Never smokers	ref	ref	ref	ref	ref
Former smokers	n/a	1.18 (0.78 - 1.79)	1.30 (1.00 - 1.69)	1.25 (0.98 - 1.59)	1.27 (1.07 - 1.49)
New current smokers	0.88 (0.36 - 2.10)	0.95 (0.62 - 1.46)	1.19 (0.91 - 1.56)	1.20 (0.96 - 1.50)	1.37 (1.19 - 1.58)
Decreasing light smokers	0.73 (0.36 - 1.48)	0.86 (0.60 - 1.25)	1.61 (1.28 - 2.03)	1.86 (1.53 - 2.27)	1.59 (1.37 - 1.84)
Steady moderate smokers	0.83 (0.46 - 1.49)	1.21 (0.88 - 1.64)	1.98 (1.60 - 2.44)	2.01 (1.66 - 2.43)	1.67 (1.42 - 1.97)
Steady heavy smokers	1.17 (0.45 - 3.02)	1.31 (0.82 - 2.10)	2.23 (1.65 - 3.03)	2.07 (1.45 - 2.96)	1.96 (1.17 - 3.29)
Colorectum					
Never smokers	ref	ref	ref	ref	ref
Former smokers	0.32 (0.04 - 2.48)	0.76 (0.45 - 1.28)	1.04 (0.80 - 1.36)	0.95 (0.74 - 1.21)	1.12 (0.94 - 1.33)
New current smokers	0.96 (0.36 - 2.54)	0.67 (0.40 - 1.15)	1.03 (0.79 - 1.34)	1.00 (0.80 - 1.24)	1.12 (0.96 - 1.29)
Decreasing light smokers	0.66 (0.28 - 1.55)	0.94 (0.63 - 1.38)	1.24 (0.99 - 1.56)	1.22 (0.99 - 1.49)	1.36 (1.17 - 1.59)
Steady moderate smokers	0.49 (0.23 - 1.04)	0.98 (0.69 - 1.39)	1.09 (0.87 - 1.35)	1.29 (1.06 - 1.57)	1.36 (1.14 - 1.62)
Steady heavy smokers	1.31 (0.47 - 3.62)	0.87 (0.49 - 1.56)	1.19 (0.83 - 1.69)	1.12 (0.74 - 1.70)	1.34 (0.71 - 2.52)
Liver					

Never smokers	ref	ref	ref	ref	ref
Former smokers	0.82 (0.28 - 2.40)	0.76 (0.52 - 1.12)	1.07 (0.89 - 1.30)	1.12 (0.95 - 1.33)	1.14 (0.97 - 1.33)
New current smokers	0.97 (0.42 - 2.19)	1.30 (0.95 - 1.77)	1.30 (1.08 - 1.55)	1.13 (0.97 - 1.33)	1.12 (0.98 - 1.28)
Decreasing light smokers	1.14 (0.62 - 2.07)	1.38 (1.06 - 1.79)	2.00 (1.72 - 2.33)	1.43 (1.23 - 1.66)	1.41 (1.23 - 1.62)
Steady moderate smokers	0.99 (0.57 - 1.70)	1.83 (1.45 - 2.31)	2.13 (1.84 - 2.46)	1.65 (1.44 - 1.90)	1.55 (1.33 - 1.80)
Steady heavy smokers	1.59 (0.69 - 3.66)	0.88 (0.57 - 1.38)	1.74 (1.38 - 2.21)	1.33 (0.99 - 1.80)	1.45 (0.85 - 2.47)
Pancreas					
Never smokers	ref	ref	ref	ref	ref
Former smokers	0.62 (0.07 - 5.18)	0.85 (0.50 - 1.46)	0.80 (0.60 - 1.05)	1.02 (0.82 - 1.26)	1.08 (0.89 - 1.32)
New current smokers	0.78 (0.16 - 3.89)	0.57 (0.31 - 1.04)	0.96 (0.74 - 1.25)	0.97 (0.80 - 1.19)	1.00 (0.84 - 1.18)
Decreasing light smokers	0.75 (0.23 - 2.47)	1.03 (0.68 - 1.56)	1.03 (0.82 - 1.30)	1.24 (1.03 - 1.50)	1.13 (0.94 - 1.36)
Steady moderate smokers	1.05 (0.40 - 2.75)	1.18 (0.82 - 1.71)	1.31 (1.07 - 1.61)	1.33 (1.11 - 1.59)	1.63 (1.35 - 1.97)
Steady heavy smokers	0.75 (0.14 - 3.92)	1.51 (0.89 - 2.57)	1.65 (1.21 - 2.26)	1.93 (1.41 - 2.65)	1.32 (0.65 - 2.66)
Larynx					
Never smokers	ref	ref	ref	ref	ref
Former smokers	n/a	n/a	n/a	0.32 (0.04 - 2.64)	1.44 (0.55 - 3.80)
New current smokers	n/a	n/a	n/a	0.94 (0.27 - 3.34)	2.00 (0.91 - 4.41)
Decreasing light smokers	n/a	n/a	n/a	1.39 (0.47 - 4.17)	3.44 (1.64 - 7.23)
Steady moderate smokers	n/a	n/a	n/a	4.03 (1.63 - 9.96)	3.51 (1.59 - 7.74)
Steady heavy smokers	n/a	n/a	n/a	6.16 (1.82 - 20.82)	7.32 (1.58 - 33.9)
Lung					
Never smokers	ref	ref	ref	ref	ref
Former smokers	0.72 (0.16 - 3.23)	0.69 (0.38 - 1.24)	1.33 (1.06 - 1.66)	1.59 (1.33 - 1.89)	2.20 (1.97 - 2.46)
New current smokers	1.65 (0.66 - 4.12)	1.43 (0.91 - 2.25)	1.25 (1.00 - 1.58)	2.06 (1.77 - 2.40)	2.11 (1.91 - 2.34)
Decreasing light smokers	1.12 (0.50 - 2.50)	1.26 (0.85 - 1.87)	1.84 (1.52 - 2.23)	3.80 (3.32 - 4.36)	4.30 (3.92 - 4.73)
Steady moderate smokers	0.82 (0.39 - 1.73)	1.94 (1.38 - 2.75)	3.52 (2.97 - 4.17)	6.77 (5.96 - 7.69)	6.70 (6.10 - 7.36)
Steady heavy smokers	1.06 (0.33 - 3.46)	3.01 (1.94 - 4.68)	5.98 (4.88 - 7.34)	10.48 (8.9 - 12.35)	10.07 (8.31 - 12.19)
Kidney					
Never smokers	ref	ref	ref	ref	ref
Former smokers	n/a	1.34 (0.42 - 4.23)	0.66 (0.31 - 1.40)	0.94 (0.56 - 1.56)	0.99 (0.64 - 1.54)
New current smokers	0.75 (0.08 - 7.31)	1.28 (0.41 - 4.03)	0.73 (0.35 - 1.49)	0.64 (0.38 - 1.09)	0.94 (0.64 - 1.37)
Decreasing light smokers	n/a	1.47 (0.57 - 3.81)	1.02 (0.57 - 1.83)	0.75 (0.45 - 1.24)	1.27 (0.85 - 1.90)
Steady moderate smokers	0.17 (0.02 - 1.72)	2.00 (0.85 - 4.69)	1.13 (0.66 - 1.92)	1.21 (0.79 - 1.86)	1.35 (0.85 - 2.15)
Steady heavy smokers	1.03 (0.09 - 11.47)	0.98 (0.20 - 4.77)	0.86 (0.32 - 2.30)	1.08 (0.42 - 2.76)	0.92 (0.13 - 6.69)
Bladder					
Never smokers	ref	ref	ref	ref	ref
Former smokers	n/a	1.78 (0.11 - 28.69)	0.90 (0.26 - 3.07)	1.40 (0.80 - 2.47)	1.33 (0.95 - 1.88)
New current smokers	n/a	3.47 (0.31 - 38.30)	0.89 (0.26 - 3.03)	1.24 (0.73 - 2.12)	1.40 (1.04 - 1.87)
Decreasing light smokers	n/a	7.49 (0.93 - 60.26)	2.60 (1.07 - 6.29)	1.64 (0.99 - 2.71)	1.74 (1.28 - 2.37)

Steady moderate smokers	n/a	2.39 (0.26 - 21.60)	2.49 (1.06 - 5.85)	2.04 (1.28 - 3.26)	2.41 (1.74 - 3.33)
Steady heavy smokers	n/a	3.36 (0.20 - 55.14)	3.07 (0.95 - 9.92)	1.74 (0.67 - 4.56)	2.03 (0.64 - 6.45)
Leukemia					
Never smokers	ref	ref	ref	ref	ref
Former smokers	1.64 (0.43 - 6.22)	0.71 (0.35 - 1.46)	1.25 (0.78 - 2.01)	0.95 (0.59 - 1.54)	0.84 (0.58 - 1.23)
New current smokers	4.18 (1.73 - 10.14)	1.02 (0.56 - 1.85)	1.29 (0.81 - 2.05)	1.51 (1.03 - 2.21)	1.20 (0.90 - 1.61)
Decreasing light smokers	0.95 (0.34 - 2.63)	0.71 (0.41 - 1.23)	1.28 (0.83 - 1.96)	1.33 (0.89 - 1.98)	1.20 (0.87 - 1.66)
Steady moderate smokers	1.55 (0.68 - 3.51)	0.69 (0.42 - 1.12)	1.13 (0.75 - 1.70)	1.82 (1.27 - 2.61)	1.35 (0.94 - 1.94)
Steady heavy smokers	2.32 (0.68 - 7.93)	0.76 (0.33 - 1.77)	1.26 (0.64 - 2.50)	1.16 (0.49 - 2.71)	0.54 (0.07 - 3.85)
Others					
Never smokers	ref	ref	ref	ref	ref
Former smokers	1.83 (0.80 - 4.19)	0.89 (0.62 - 1.28)	1.05 (0.87 - 1.26)	0.99 (0.85 - 1.15)	1.02 (0.91 - 1.13)
New current smokers	1.26 (0.60 - 2.66)	1.12 (0.81 - 1.56)	0.93 (0.76 - 1.13)	0.95 (0.83 - 1.10)	1.03 (0.94 - 1.14)
Decreasing light smokers	1.93 (1.10 - 3.37)	0.94 (0.71 - 1.26)	0.97 (0.81 - 1.15)	1.08 (0.95 - 1.24)	1.17 (1.06 - 1.29)
Steady moderate smokers	1.10 (0.64 - 1.90)	0.97 (0.75 - 1.26)	1.11 (0.95 - 1.30)	1.21 (1.06 - 1.37)	1.19 (1.07 - 1.34)
Steady heavy smokers	1.10 (0.43 - 2.81)	1.05 (0.70 - 1.59)	1.27 (0.98 - 1.64)	1.13 (0.86 - 1.49)	1.39 (0.95 - 2.04)

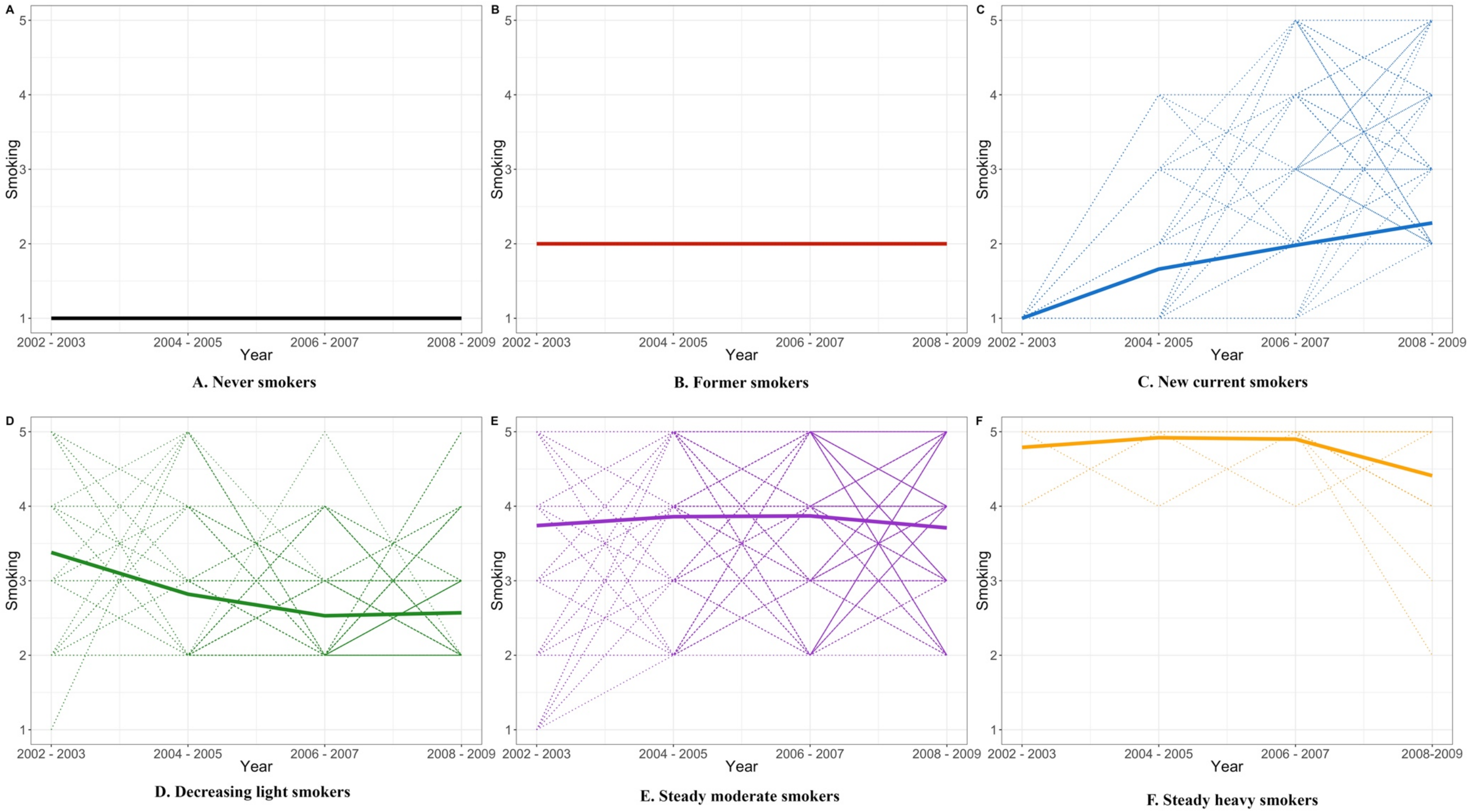

Appendix Figure 1. Smoking pattern of participants from 2002-2009 by smoking trajectory groups.

Cancer Site	Cases	Adjusted Hazard ratio (95% CI)
**All cancer**		
Never smokers	29007	ref
Former smokers	16966	1.10 (1.08 - 1.13)
New current smokers	21654	1.13 (1.11 - 1.15)
Decreasing light smokers	28012	1.30 (1.28 - 1.33)
Steady moderate smokers	36653	1.45 (1.43 - 1.48)
Steady heavy smokers	5404	1.53 (1.49 - 1.58)
**Lip, oral cavity and pharynx**		
Never smokers	346	ref
Former smokers	220	1.15 (0.97 - 1.37)
New current smokers	275	1.21 (1.03 - 1.42)
Decreasing light smokers	394	1.40 (1.20 - 1.62)
Steady moderate smokers	636	1.87 (1.62 - 2.14)
Steady heavy smokers	100	1.99 (1.58 - 2.51)
**Esophagus**		
Never smokers	260	ref
Former smokers	224	1.50 (1.25 - 1.80)
New current smokers	255	1.37 (1.15 - 1.63)
Decreasing light smokers	404	1.91 (1.63 - 2.25)
Steady moderate smokers	652	2.84 (2.44 - 3.31)
Steady heavy smokers	104	3.10 (2.43 - 3.95)
**Stomach**		
Never smokers	5075	ref
Former smokers	3334	1.23 (1.18 - 1.29)
New current smokers	4207	1.25 (1.20 - 1.30)
Decreasing light smokers	5594	1.47 (1.42 - 1.53)
Steady moderate smokers	7607	1.69 (1.63 - 1.76)
Steady heavy smokers	1122	1.78 (1.66 - 1.90)
**Colorectum**		
Never smokers	4084	ref
Former smokers	2520	1.15 (1.09 - 1.21)
New current smokers	3084	1.14 (1.08 - 1.19)
Decreasing light smokers	3955	1.28 (1.22 - 1.34)
Steady moderate smokers	4935	1.34 (1.28 - 1.40)
Steady heavy smokers	683	1.29 (1.19 - 1.41)
**Liver**		
Never smokers	2244	ref
Former smokers	1248	1.03 (0.96 - 1.11)
New current smokers	1790	1.17 (1.10 - 1.25)
Decreasing light smokers	2505	1.52 (1.43 - 1.61)
Steady moderate smokers	3307	1.72 (1.63 - 1.82)
Steady heavy smokers	357	1.31 (1.17 - 1.47)
**Pancreas**		
Never smokers	762	ref
Former smokers	433	1.08 (0.96 - 1.22)
New current smokers	557	1.08 (0.96 - 1.20)
Decreasing light smokers	681	1.25 (1.12 - 1.39)
Steady moderate smokers	955	1.63 (1.47 - 1.80)
Steady heavy smokers	160	2.00 (1.67 - 2.40)
**Larynx**		
Never smokers	91	ref
Former smokers	101	2.07 (1.55 - 2.78)
New current smokers	124	1.95 (1.48 - 2.58)
Decreasing light smokers	222	3.35 (2.61 - 4.32)
Steady moderate smokers	426	5.87 (4.62 - 7.46)
Steady heavy smokers	91	9.57 (7.03 - 13.01)
**Lung**		
Never smokers	1796	ref
Former smokers	1528	1.68 (1.56 - 1.80)
New current smokers	2086	1.65 (1.55 - 1.76)
Decreasing light smokers	3550	2.97 (2.80 - 3.15)
Steady moderate smokers	5800	4.90 (4.64 - 5.18)
Steady heavy smokers	1043	7.45 (6.88 - 8.08)
**Kidney**		
Never smokers	945	ref
Former smokers	492	0.97 (0.86 - 1.08)
New current smokers	598	1.02 (0.92 - 1.14)
Decreasing light smokers	765	1.01 (0.91 - 1.11)
Steady moderate smokers	1067	1.07 (0.98 - 1.17)
Steady heavy smokers	173	1.15 (0.97 - 1.36)
**Bladder**		
Never smokers	824	ref
Former smokers	583	1.38 (1.24 - 1.54)
New current smokers	842	1.50 (1.36 - 1.65)
Decreasing light smokers	960	1.76 (1.60 - 1.94)
Steady moderate smokers	1255	2.19 (1.99 - 2.40)
Steady heavy smokers	189	2.64 (2.24 - 3.11)
**Leukemia**		
Never smokers	427	ref
Former smokers	221	0.99 (0.84 - 1.17)
New current smokers	341	1.28 (1.11 - 1.48)
Decreasing light smokers	390	1.18 (1.03 - 1.36)
Steady moderate smokers	494	1.18 (1.03 - 1.35)
Steady heavy smokers	75	1.23 (0.95 - 1.59)
**Others**		
Never smokers	12153	ref
Former smokers	6062	0.94 (0.92 - 0.97)
New current smokers	7495	0.96 (0.93 - 0.99)
Decreasing light smokers	8592	0.94 (0.91 - 0.97)
Steady moderate smokers	9519	0.86 (0.83 - 0.88)
Steady heavy smokers	1307	0.83 (0.78 - 0.88)

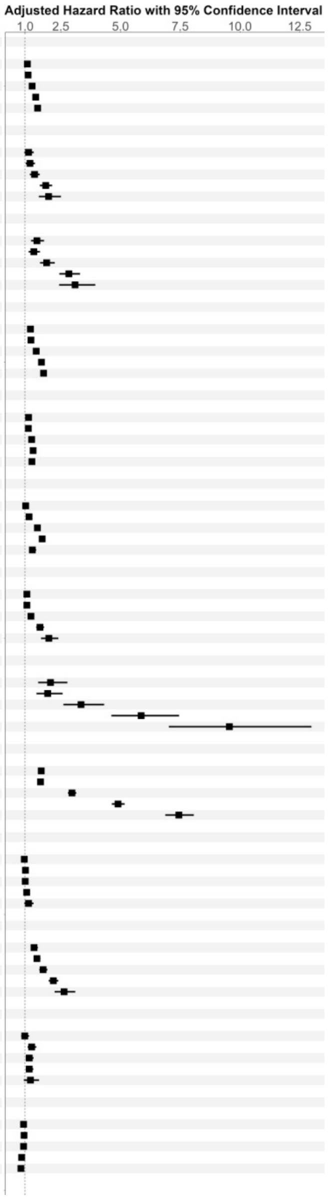

Appendix Figure 2. Smoking trajectory and cancer incidence (Cox proportional hazards model)

Cancer Site	Death	Adjusted Hazard ratio (95% CI)
**All cancer**		
Never smokers	7479	ref
Former smokers	4560	1.17 (1.13 - 1.21)
New current smokers	6600	1.22 (1.17 - 1.26)
Decreasing light smokers	8837	1.68 (1.63 - 1.73)
Steady moderate smokers	11907	2.22 (2.15 - 2.29)
Steady heavy smokers	1725	2.64 (2.50 - 2.79)
**Lip, oral cavity and pharynx**		
Never smokers	91	ref
Former smokers	57	1.16 (0.83 - 1.64)
New current smokers	88	1.36 (1.00 - 1.83)
Decreasing light smokers	114	1.65 (1.24 - 2.20)
Steady moderate smokers	201	2.66 (2.04 - 3.46)
Steady heavy smokers	38	3.88 (2.60 - 5.79)
**Esophagus**		
Never smokers	130	ref
Former smokers	107	1.44 (1.11 - 1.87)
New current smokers	126	1.27 (0.98 - 1.63)
Decreasing light smokers	220	2.00 (1.60 - 2.50)
Steady moderate smokers	320	2.79 (2.25 - 3.47)
Steady heavy smokers	49	3.36 (2.37 - 4.76)
**Stomach**		
Never smokers	795	ref
Former smokers	500	1.23 (1.10 - 1.38)
New current smokers	714	1.27 (1.14 - 1.40)
Decreasing light smokers	900	1.57 (1.42 - 1.73)
Steady moderate smokers	1106	1.85 (1.68 - 2.04)
Steady heavy smokers	163	2.17 (1.81 - 2.59)
**Colorectum**		
Never smokers	801	ref
Former smokers	436	1.02 (0.91 - 1.15)
New current smokers	609	1.04 (0.93 - 1.16)
Decreasing light smokers	720	1.26 (1.14 - 1.40)
Steady moderate smokers	790	1.29 (1.16 - 1.43)
Steady heavy smokers	103	1.33 (1.08 - 1.65)
**Liver**		
Never smokers	1230	ref
Former smokers	702	1.07 (0.97 - 1.18)
New current smokers	1014	1.17 (1.07 - 1.27)
Decreasing light smokers	1425	1.56 (1.44 - 1.68)
Steady moderate smokers	1880	1.82 (1.68 - 1.96)
Steady heavy smokers	203	1.45 (1.24 - 1.69)
**Pancreas**		
Never smokers	766	ref
Former smokers	389	0.97 (0.86 - 1.10)
New current smokers	511	0.95 (0.85 - 1.06)
Decreasing light smokers	633	1.15 (1.03 - 1.28)
Steady moderate smokers	861	1.48 (1.33 - 1.64)
Steady heavy smokers	144	1.90 (1.58 - 2.29)
**Larynx**		
Never smokers	17	ref
Former smokers	10	1.19 (0.54 - 2.62)
New current smokers	24	1.81 (0.95 - 3.44)
Decreasing light smokers	39	3.17 (1.76 - 5.71)
Steady moderate smokers	60	4.91 (2.77 - 8.72)
Steady heavy smokers	14	11.51 (5.43 - 24.39)
**Lung**		
Never smokers	1162	ref
Former smokers	1074	1.80 (1.66 - 1.96)
New current smokers	1661	1.91 (1.77 - 2.06)
Decreasing light smokers	2852	3.66 (3.42 - 3.93)
Steady moderate smokers	4479	6.34 (5.92 - 6.78)
Steady heavy smokers	736	9.94 (9.02 - 10.95)
**Kidney**		
Never smokers	149	ref
Former smokers	69	0.79 (0.56 - 1.12)
New current smokers	89	0.89 (0.70 - 1.13)
Decreasing light smokers	101	0.99 (0.73 - 1.34)
Steady moderate smokers	144	1.35 (1.05 - 1.73)
Steady heavy smokers	15	1.09 (0.62 - 1.91)
**Bladder**		
Never smokers	126	ref
Former smokers	81	1.30 (0.98 - 1.72)
New current smokers	129	1.34 (1.05 - 1.73)
Decreasing light smokers	147	1.87 (1.47 - 2.40)
Steady moderate smokers	157	2.34 (1.83 - 3.01)
Steady heavy smokers	14	2.20 (1.25 - 3.87)
**Leukemia**		
Never smokers	238	ref
Former smokers	112	0.92 (0.73 - 1.15)
New current smokers	213	1.31 (1.08 - 1.58)
Decreasing light smokers	192	1.12 (0.92 - 1.37)
Steady moderate smokers	255	1.28 (1.06 - 1.54)
Steady heavy smokers	32	1.15 (0.77 - 1.70)
**Others**		
Never smokers	1974	ref
Former smokers	1023	0.99 (0.92 - 1.08)
New current smokers	1422	1.00 (0.93 - 1.07)
Decreasing light smokers	1494	1.10 (1.03 - 1.18)
Steady moderate smokers	1654	1.21 (1.13 - 1.30)
Steady heavy smokers	214	1.27 (1.10 - 1.48)

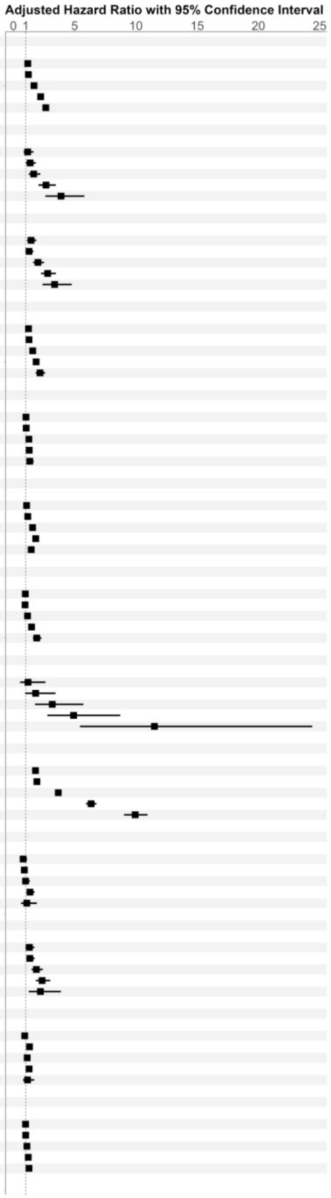

Appendix Figure 3. Smoking trajectory and cancer mortality (Cox proportional hazards model)

© 2022 Luu M.N. et al.
